# Characterization of C5 Acylcarnitines and Related Dicarboxylic Acylcarnitines in Saudi Newborns: Screening, Confirmation, and Cutoff Variation

**DOI:** 10.3390/ijns11020036

**Published:** 2025-05-12

**Authors:** Hanadi A. Bokhari, Ahmed H. Mujamammi, Huda A. Bader, Hannadi J. Alamri, Khalid K. Alharbi

**Affiliations:** 1 Newborn Screening Laboratory, Public Health Authority, Riyadh 13351, Saudi Arabia; habokhari88@gmail.com (H.A.B.); hudabader.85@gmail.com (H.A.B.); hannadi.alamri@gmail.com (H.J.A.); 2 Clinical Biochemistry Unit, Department of Pathology, College of Medicine, King Saud University, Riyadh 11461, Saudi Arabia; 3 Department of Clinical Laboratory Sciences, College of Applied Medical Sciences, King Saud University, Riyadh 11433, Saudi Arabia; kharbi@ksu.edu.sa

**Keywords:** newborn screening, disability, Saudi population, C5 acylcarnitine

## Abstract

Newborn screening (NBS) is a nationwide program for the early detection of disability in the Saudi population. This study focused on specific disorders related to organic acids that share C5 acylcarnitines derivatives and related dicarboxylic acylcarnitines as primary screening metabolites. We aimed to determine the frequency of C5 acylcarnitine derivatives and related dicarboxylic acylcarnitines among screened newborns; confirm truly positive screening results using urine organic acid analysis; and compare the cutoff values for C5, C5DC, and C5OH acylcarnitines from the selected analytical centers. Data from laboratory positively screened and confirmed samples from the Public Health Authority (PHA) over 3 years were retrieved and analyzed to determine the frequency of the selected metabolites and percentage of true positive results among the positively screened samples. We identified significant correlations among variables such as disease, sex, and C5 metabolites across different cities. We clarified the frequency of true positive results for C5 acylcarnitine derivatives and related dicarboxylic acylcarnitines among Saudi newborns and highlighted significant variations in cutoff values across analytical centers. These findings contribute to the enhancement of NBS protocols and early intervention strategies.

## 1. Introduction

Newborn screening (NBS) programs worldwide mark a pivotal advancement in public health striving to promptly intercept and manage congenital disorders [1]. In the United States and globally, the number of rare conditions for which newborns are screened has increased over time, and currently, most states screen approximately 51 conditions [2]. These conditions were recommended by the National Newborn Screening and Genetics Resource Center. In Saudi Arabia, the Ministry of Health (MOH) continues to implement a national NBS program to early intervention for congenital disorders, reducing the potential for severe disabilities. The Saudi Arabian government introduced the National Transformation Program in 2016 to ensure the realization of Vision 2030 [3]. Advancements in science and technology have led to a dramatic increase in the number of conditions screened for in NBS programs, from an average of one to over 50 tests. This national trend of expanding NBS programs has outpaced the development of adequate healthcare infrastructure for managing identified cases, resulting in significant ethical, social, and legal challenges [2]. NBS programs are crucial for the 500,000 Saudi babies born in the Kingdom of Saudi Arabia each year. This is a coordinated and all-encompassing system that includes program evaluation, education, screening, diagnosis, treatment, and management [4]. This program was designed for early detection of genetic disorders that cause severe complications across the Kingdom.

In Saudi Arabia, NBS plays a crucial role because of the distinct genetic landscape characterized by high rates of consanguineous marriages, which substantially increases the incidence of recessive genetic disorders [5]. These genetic factors necessitate a robust screening program to prevent severe disability and mortality due to metabolic disorders that are otherwise asymptomatic during the newborn period. The Middle East and North Africa (MENA) region consists of 21 countries with a population of approximately 400 million people and an estimated 10 million newborns annually. There are significant differences among countries in terms of population size, per capita income, healthcare systems, insurance coverage, and different stages of epidemiologic transition. As the population in the region is characterized by high consanguinity and marriages among first cousins, genetic disorders are relatively common [6].

Acylcarnitines play a pivotal role in fatty acid metabolism, which is a critical biochemical pathway for energy production, especially during periods of fasting or increased energy demand. C5 acylcarnitine is an extremely valuable marker in NBS because it contains more than one derivative, each of which is considered an indicator of a particular disease. These diseases have an impact on the life of infants if not detected early. From the Saudi NBS panel, an elevation of C5 is indicative of the isovaleric acidemia (IVA) and high C5-DC level is suggestive of newborn with Glutaric Aciduria Type 1 (GA1), while C5OH elevation is suggestive of 3-Hydroxy-3-Methylglutaraciduria (3HMG), 3-Methylcrotonylglycinuria (3MCC), or Beta-ketothiolase deficiency (βKT). Leucine is broken down, producing isovaleryl-CoA and 3-hydroxyisovaleryl-CoA through a series of enzymatic reactions within the mitochondria. These intermediate acyl-CoA molecules are then conjugated with carnitine. This conjugation, forming isovalerylcarnitine (C5) and 3-hydroxyisovalerylcarnitine (C5OH), is crucial for transporting these compounds out of the mitochondria. Specifically, isovaleryl-CoA combines with carnitine to make C5, and 3-hydroxyisovaleryl-CoA combines with carnitine to make C5OH. The levels of these carnitine derivatives provide insight into the efficiency and functionality of leucine metabolism. Glutarylcarnitine (C5DC) is a crucial indicator of lysine and tryptophan metabolism, especially in diagnosing glutaric acidemia type 1 (GA-1). Lysine’s breakdown produces glutaryl-CoA, and a deficiency in glutaryl-CoA dehydrogenase (GCDH) prevents its proper breakdown, leading to its accumulation. Tryptophan’s metabolic pathways also contribute to glutaryl-CoA production. When glutaryl-CoA builds up, it is conjugated with carnitine to form C5DC, a process the body uses to remove excess acyl-CoA from mitochondria. Therefore, elevated C5DC levels signal the accumulation of glutaryl-CoA, indicating a potential metabolic disorder [7].

In Saudi Arabia, NBS tests all babies soon after birth (within 24–72 h) for certain disorders, including amino acidopathies, fatty acid oxidation disorders, organic acidemias, and endocrine and enzymatic problems; that is [8], all babies are subjected to NBS, a series of tests that look for specific uncommon disorders. Multiple genetic and metabolic abnormalities are associated with certain medical problems. Blood tests were conducted in the laboratory. NBS involves conducting a group of tests from a dried blood spot (DBS), which is obtained by pricking the newborn’s heel and obtaining a few drops of blood on a specific filter paper [9]. Therefore, tandem mass spectrometry is used in the NBS test to provide a reliable measure of specificity and sensitivity in examination results, where only one metabolite can be used for the simultaneous identification of multiple disorders at the same time. The main purpose of this screening is to aid in the early detection of certain disorders that could otherwise not be identified before the birth of a newborn. Early detection of these conditions would help doctors in the treatment and management and promote the health of newborns. Therefore, it is essential to increase infant survival and ensure good health conditions in the future.

From the Saudi NBS panel, NBS for isovaleric acidemia (IVA), glutaric aciduria type 1 (GA1), 3-hydroxy-3-methylglutaraciduria (3HMG), 3-methylcrotonyl-CoA carboxylase deficiency (3MCC), and β-ketothiolase deficiency (βKT) involves analyzing DBS via liquid chromatography–tandem mass spectrometry (LC-MS/MS). Elevated C5 indicates IVA (cutoff 0.7), elevated C5DC+C6OH indicates GA1 (cutoff 0.6), and elevated C5OH indicates 3HMG/3MCC (cutoff 0.8). βKT is identified by elevated C5:1 (cutoff 0.15) and C5OH (cutoff 0.8). Positive screens require follow-up, with confirmatory urine organic acid (UOA) analysis using gas chromatography/mass spectrometry (GC/MS). UOA reveals elevated 3HIA and IVG in IVA, glutaric acid and 3-hydroxyglutaric acid in GA1, 3HIA, 3-methylglutaconic acid, 3HMG, 3-MG-A, and sometimes 3-methylcrotonylglycine in 3HMG, 3-MCG, 3HIV-A, and IVG in 3MCC, and 3-hydroxybutyric acid, 2-methyl-3-hydroxybutyric acid, and tiglylglycine in βKT [8,10].

The implementation of the NBS program in Saudi Arabia is not only a preventive measure but also a necessary intervention, given the high risk of inherited metabolic disorders. The focus of this study on C5 acyl carnitine and its derivatives is particularly relevant, given the spectrum of organic acids prevalent in the region. By analyzing the frequency of these metabolites and exploring the variability in cutoff values among different testing centers, this study aimed to enhance the screening accuracy and ensure that affected newborns receive prompt and effective treatment. This study provides significant insights into the effectiveness of NBS in Saudi Arabia. These insights have the potential to guide future enhancements to both the screening process and broader public health strategies aimed at mitigating the burden of genetic disorders within the population. The outcomes of this study hold profound implications for health policy and the management of congenital disorders in regions with similar genetic predispositions. This study focuses on the characterization of isovalerylcarnitine (C5), 3-hydroxyisovalerylcarnitine (C5OH), and glutarylcarnitine (C5DC), which are associated with isovaleric acidemia (IVA), 3-methylcrotonyl-CoA carboxylase deficiency (3-MCCD), and glutaric acidemia type 1 (GA1), respectively. This study aims to enhance the analytical characterization of these metabolites, thereby improving the accuracy and efficiency of NBS for these critical disorders. The aim of the study is (1) to determine the frequency of C5 acylcarnitine derivatives and related dicarboxylic acylcarnitines among screened newborns in Saudi Arabia; (2) to confirm truly positive screening results using urine organic acid analysis and calculate the positive predictive value (PPV) for each disorder; (3) to analyze the distribution of organic acid disorders and related C5 metabolites across different cities and genders in Saudi Arabia; (4) to evaluate the concordance between LC-MS/MS screening and urine organic acid confirmation for metabolic disorders and to compare the cutoff values for C5, C5DC, and C5OH acylcarnitines across different analytical centers to assess inter-laboratory variability.

## 2. Materials and Methods

### 2.1. Ethical Approval

The study was approved by the Institutional review board approval was obtained from Public Health Authority, Saudi Arabia (IRB Number: SCDC-IRB-A057-2023). The study was conducted in accordance with the ethical standards of the Declaration of Helsinki.

### 2.2. Study Population and Data Collection

This observational cohort study encompassed NBS data of Saudi population with elevated levels of primary acylcarnitines (C5, C5DC, C5OH) and secondary marker C5:1, collected from 2020 to 2022 at the Public Health Authority Laboratory in Saudi Arabia. This study examined Saudi newborns aged between 1 and 72 h, comprising 213 (46.8%) males and 242 (53.2%) females. This laboratory serves both government and private hospitals nationwide. In this study, all laboratory-positive NBS results with high levels of primary markers C5, C5DC, and C5OH acylcarnitines and secondary marker C5: 1 by DBS screening liquid chromatography–tandem mass spectrometry and urine organic acid analysis using gas chromatography–mass spectrometry (GC-MS) performed as per the previous protocol were included [11,12,13,14]. Briefly, DBS samples, collected via heel prick onto barcoded Whatman 903 filter paper, were analyzed for metabolic disorder in newborns. Acylcarnitine analysis was performed using liquid chromatography–tandem mass spectrometry (LC-MS/MS)with a Waters ACQUITY UPLC™ system coupled to MS/MS TQD (Micromass UK Limited, Manchester, UK), employing a reagent kit (Chromsystem Germany). Mass spectrometry detection utilized multiple reaction monitoring, with compound quantification based on signal intensity ratios to internal standards. Positive screening results were reviewed by a multidisciplinary hospital committee and communicated to healthcare providers for follow-up diagnostic testing, adhering to Ministry of Health guidelines.

The exclusion criteria included unremarkable NBS results or positive NBS results with acylcarnitines other than the primary markers C5, C5DC, and C5OH acylcarnitines and secondary marker C5:1. The dataset for this study was compiled from records, including demographic information, screening results from liquid chromatography–tandem mass spectrometry, and confirmation via urine organic acid tests, with a focus on the selection of metabolic disorders. The retention times of metabolites were determined by gas chromatography–mass spectrometry (GC-MS) (Agilent Technology, USA) and are illustrated in Figures S1–S5.

### 2.3. Comparison of the Cutoffs Among Selected Hospitals

The cutoff values for metabolites C5, C5DC, and C5OH across the four healthcare facilities were analyzed. Given the specific nature of these cutoff values, a descriptive approach was adopted, with visual comparisons made across institutions. Each hospital was treated as a separate entity with unique testing protocols, and these values were assumed to be the optimal operational values derived from internal standardization. For the analysis, we had chosen cutoff values for each metabolite from the Public Health Laboratory (PHL). According to PHL Isovaleric acidemia (IVA), the cutoff level of C5 is (0.7).

Glutaric aciduria type 1 (GA1): the cutoff level for (C5DC + C6OH) is (0.6); 3-hydroxy-3-methylglutaraciduria (3HMG): the cutoff level of C5OH is (0.8); methylcrotonylglycinuria (3MCC): the cutoff level of C5OH is (0.8); βeta-ketothiolase deficiency (βKT): the cutoff level for (C5:1) is (0.15) and (C4DC + C5OH) is (0.8).

### 2.4. Statistical Analysis

Data analysis was conducted using the Statistical Package for the Social Sciences (SPSS) software (version 25.0; IBM Corp., Armonk, NY, USA). Descriptive statistics, including frequencies and distributions, were computed to summarize the data across genders and cities. The number of positive MS/MS results was compared with those confirmed by urine organic acid tests for each disorder. Pearson’s correlation coefficient was calculated to evaluate the consistency between the two diagnostic methods. Visualizations, including bar graphs and heat maps, were created using SPSS software to display the distribution of disorders and discrepancies in the diagnostic results.

## 3. Results

Out of a total of 822,887 NBS results during this period (256,033 in 2020, 300,161 in 2021, and 266,693 in 2022), 822,432 (99.9%) showed unremarkable results for the specified acyl carnitines urine organic acid (UOAs) analysis confirmed 455 true positive cases out of 1604 samples that initially showed elevated primary markers in NBS.

### 3.1. Yearly Distribution of Metabolites

**Figure 1:**
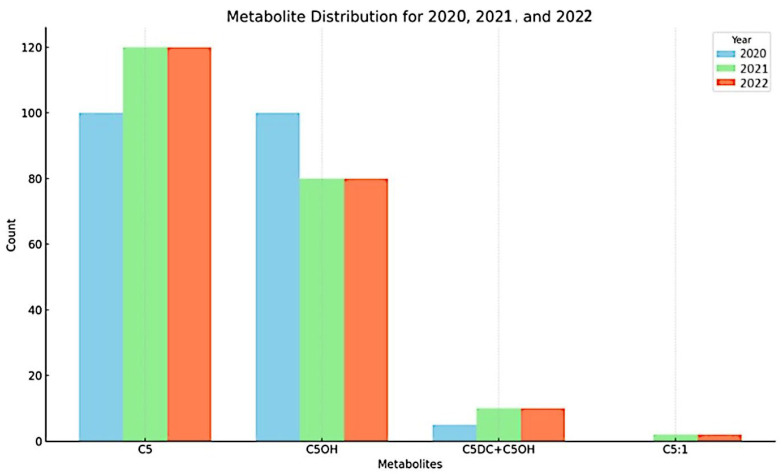
Distribution of metabolites across years.

The yearly distribution of metabolites in 2020, 2021, and 2022, as shown in Figure 1, reveals distinct patterns across the three years. In 2020 (light blue bars), metabolites C5 and C5OH exhibited the highest occurrence, with both present at approximately equal levels. In 2021 (green bars), the distribution shifted. While C5 remained prominent with an increased level, C5OH showed a decrease compared to 2020. Notably, metabolites C5DC + C6OH and C5:1, which had very low or negligible presence in 2020, were detected, indicating an increase in metabolite diversity. By 2022 (red bars), C5 continued to be the most frequently occurring metabolite. C5OH remained at a lower level than C5, and C5DC + C6OH was detected at a low level. Overall, the figure illustrates that C5 and C5OH were the primary metabolites detected across the three years. C5 consistently exhibited a high presence, increasing from 2020 to 2022. While C5OH was also high in 2020, its levels decreased in subsequent years. Additionally, the presence of C5DC + C6OH and C5:1 increased over the period, although their overall levels were considerably lower than C5 and C5OH. These patterns suggest a dynamic change in metabolite profiles from 2020 to 2022.

### 3.2. False Positives by Disorder

Analysis of the screening tests revealed a significant discrepancy in the number of false positives across various disorders when comparing MS/MS results and urine organic acid confirmations. 3-Hydroxy-3-Methylglutaraciduria (3HMG)/3MCC showed the highest number of false positives (99), indicating potential over detection by MS/MS. The IVA also had a substantial number of false positives, totaling 83. Fewer discrepancies were observed in Glutaric Aciduria Type 1 (GA1) and BKT, with four and three false positives, respectively, suggesting better alignment between the testing methods for these disorders (Table 1).

### 3.3. Concordance Between LC-MS/MS Screening and UOA Confirmation for Metabolic Disorders

**Figure 2:**
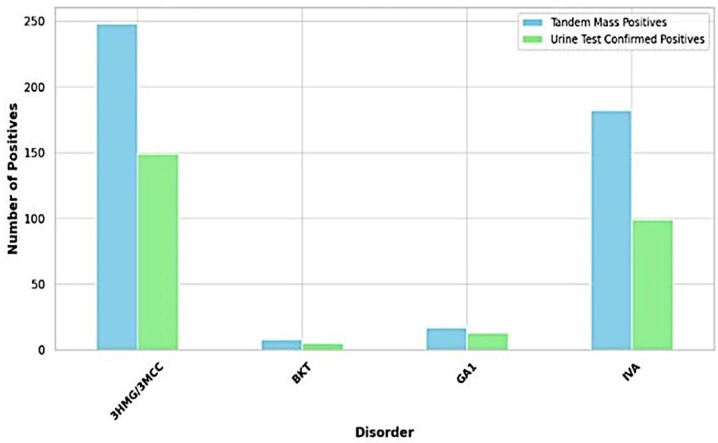
Concordance between LC-MS/MS Screening and UOA Confirmation for Metabolic Disorders.

The study analyzed the concordance between initial screening results using LC-MS/MS and confirmatory urine organic acid tests (UOA) across different metabolic disorders. It is important to note that the following true positive rates reflect the proportion of cases identified by LC-MS/MS that were subsequently confirmed by UOA within the sample set analyzed and are not relativized to overall birth numbers or general population prevalence. For 3HMG/3MCC, LC-MS/MS identified 248 cases, with UOA confirming 149 (true positive rate: 60.1%). In the case of IVA, LC-MS/MS detected 182 cases, with UOA confirming 99 (true positive rate: 54.4%). For GA1, LC-MS/MS detected 17 cases, with UOA confirming 13 (true positive rate: 76.5%). Finally, for BKT, LC-MS/MS detected 8 cases, with UOA confirming 5 (true positive rate: 62.5%) Figure 2. As shown in Table 1, GA1 exhibited the highest Positive Predictive Value (PPV) at 76.47%, suggesting that a positive screening result for GA1 in this study is most likely to be accurate within this sample. Conversely, IVA showed the lowest PPV (54.40%), indicating a higher chance of a positive result being a false positive within this sample. The PPV for BKT was 62.50%.

### 3.4. Overall Distribution of Metabolites Across Cities

**Figure 3:**
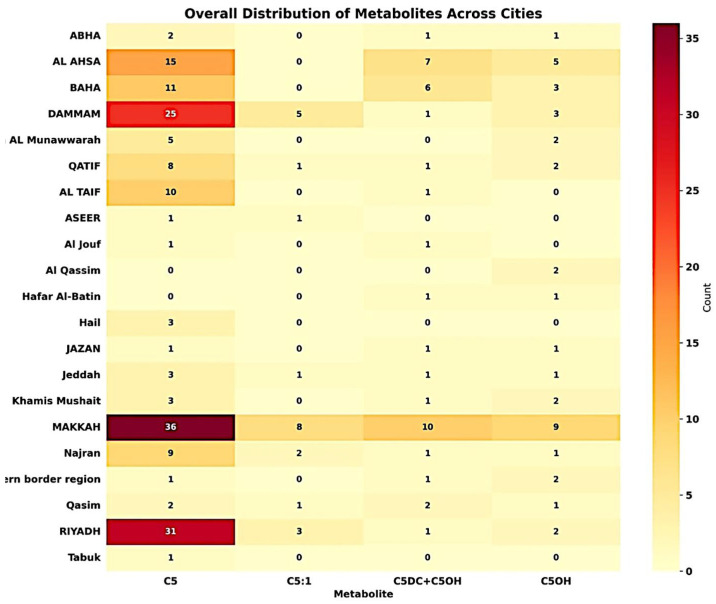
Overall distribution of metabolites across cities.

The heatmap visualization (Figure 3) outlines the distribution and frequency of various metabolites across multiple cities within this study’s dataset. Notably, cities like Jazan and AL Hassa show a higher frequency of metabolites such as C5OH, while Riyadh is characterized by a significant occurrence of both C5OH and C5. The intense shading in the heatmap for these metabolites in these cities suggests high concentrations or repeated detection over the surveyed period from 2020 to 2022 within the analyzed samples.

### 3.5. Distribution of Metabolic Disorders by City

The most prevalent disorder among the cities was 3HMG/3MCC, particularly dominant in Riyadh, Al Ahsa, Qassim and Jazan, with the highest number of cases reported in Al Ahsa. Other cities such as Jeddah, Al Madinah also showed significant numbers. The second most common disorder was IVA, with a high incidence rate in Makkah, Riyadh, and Dammam. GA1 has a lower prevalence compared to 3HMG/3MCC and IVA; however, it has been reported in multiple cities, with the highest occurrence in Riyadh, and Jazan. BKT was the least common among the disorders recorded, with scattered cases and few occurrences in cities, such as Al Baha and Jazan (Table S1).

Riyadh exhibited the highest diversity and number of metabolic disorder cases, with substantial cases of all four disorders, highlighting it as a critical region for metabolic disorder management and research. Al-Ahsa et al. reported a high number of cases of both 3HMG/3MCC, with fewer occurrences of GA1 and no reported cases of BKT. Makkah et al. reported a significant number of cases of 3HMG/3MCC and IVA, with no reports of BKT and a moderate number of GA1 cases. Jazan showed a considerable number of cases for 3HMG/3MCC and IVA, with a notable presence of GA1. Other cities, such as Al Madinah, Al Baha, and Qassim, show a variety of cases, but in smaller numbers than those in Riyadh and Makkah.

The data indicated a heterogeneous distribution of metabolic disorders across different cities, with certain disorders such as 3 HMG/3MCC and IVA being more prevalent. Riyadh, Al Ahsa, and Makkah emerged as critical regions with high incidences, necessitating focused healthcare strategies and research in these areas. Identification of these patterns is crucial for public health planning, resource allocation, and future research to understand the underlying causes of regional differences in the prevalence of metabolic disorders.

### 3.6. Cutoff Value Comparison

The cutoff values for the three metabolites, C5, C5DC, and C5OH, across the four different healthcare institutions are illustrated. Each institution has set its own threshold levels above which the presence of a metabolite is considered significant (Table 2).

The National Guard Health Affairs in Riyadh has relatively lower cutoff values for C5DC and C5OH than other institutions, whereas its cutoff for C5 is in the mid-range. Prince Sultan Military Medical City set the highest cutoff for C5OH at >0.9, indicating a more stringent criterion for this metabolite. King Faisal Specialist Hospital has consistent cutoffs for C5DC and C5OH, both >0.35 and >0.8, respectively. The Public Health Authority sets the highest cutoff for C5DC at >0.6 and maintains a cutoff of >0.8 C5 and C5OH, demonstrating a conservative approach across all three metabolites.

## 4. Discussion

The data presented in our study offer a comprehensive analysis of the spatial and temporal distributions of metabolites indicative of metabolic disorders in newborns. Our observations suggest that a variety of factors such as genetic factors, local environmental conditions, industrial outputs, and regional dietary patterns could significantly influence metabolite levels. One notable finding from our study was the particularly high detection rates of certain metabolites in specific cities, such as Jazan and AL Ahsa. This could potentially be attributed to the proximity to industrial zones where emissions of organic pollutants are prevalent or to regional dietary customs that influence metabolic profiles through the consumption of specific foods high in certain precursors or cofactors necessary for metabolic processes.

Additionally, the variability in the cutoff values used by different healthcare institutions has emerged as a significant factor affecting the detection and subsequent management of metabolic disorders. Different institutions may use assays developed by different companies based on the same method, but with varying reagent types and preparation methods, which can lead to differences in cutoff values. This variability underscores the necessity for standardized protocols to ensure consistent clinical interpretations, and the need to maintain the integrity of patient care across various health systems.

The data presented in our study offers a comprehensive analysis of the spatial and temporal distribution of metabolites indicative of metabolic disorders in newborns, with a particular focus on the variance observed in C5, C5DC, and C5OH levels across different regions and over successive years. Regarding genetic factors involvement, research highlights the significant impact of consanguineous marriages on the prevalence of genetic disorders in newborns. Studies on various populations have indicated that autosomal recessive disorders, including C5 metabolic disorders, are prevalent in regions with high rates of consanguinity. For instance, in the Saudi population, consanguineous marriages contribute to a higher incidence of these disorders [15], a finding echoed in Afghanistan, where genetic mutations associated with diseases in newborns are prevalent because of consanguinity [16]. Moreover, severe primary immunodeficiency in consanguineous unions underscores the need for early diagnosis and intervention, as evidenced by Al-Herz et al. [17]. In Iran, consanguinity has been significantly associated with higher rates of amino acid disorders in newborns, highlighting the genetic risks posed by familial marriage [18]. Furthermore, the carrier rate for hemoglobinopathies in India suggests the genetic implications of consanguinity on newborn health whereas the high prevalence of Phenylketonuria in southern Iran further illustrates this impact [19]. Additionally, Primary Congenital Glaucoma, which is prevalent among offspring of consanguineous marriages, emphasizes the broader spectrum of congenital conditions influenced by genetic factors in such unions [16].

Collectively, these findings underscore the critical need for genetic screening and counseling in populations with high rates of consanguineous marriages to mitigate the risk of metabolic and genetic disorders in newborns.

Additionally, data from our study underscore the variation in metabolite concentrations over the years, particularly noting peaks in 2020, which prompts a detailed exploration of the impact of external factors, such as changes in public health policies, environmental regulations, and even shifts in the methodologies employed in NBS programs. The year 2020, marked by significant global events, such as the COVID-19 pandemic, brought about unprecedented changes in public health responses, which could have indirectly affected screening programs, either through resource reallocation or alterations in healthcare practices. Environmental regulations may also vary significantly from year to year, influenced by policy shifts that could alter pollutant exposure or other environmental risks affecting the metabolic functions in newborns [20,21]. Moreover, advancements in screening technologies or modifications in the protocols used can lead to variations in the detected metabolite levels; newer, more sensitive technologies might identify metabolites that were previously undetectable, or changes in the protocol might affect the specificity and sensitivity of the tests. Previous studies have demonstrated that technological advancements and methodological changes can significantly influence the outcomes of metabolic screening, illustrating the dynamic nature of biochemical assessments that are responsive to both temporal and technological advancements [22].

In clinical settings, the ramifications of these findings are profound, particularly because of the observed discrepancies in the cutoff values for metabolites, such as C5, C5DC, and C5OH, across different healthcare institutions. The lack of standardization of these cutoff thresholds can lead to significant differences in the clinical management of patients, potentially resulting in over- or under-treatment. Institutions with higher cutoff values may delay necessary interventions, potentially preventing timely treatments that could avert severe outcomes. Conversely, institutions with lower thresholds could initiate treatments for false positives, leading to unnecessary medical interventions and associated healthcare costs as well as emotional stress for families. This variability in cutoff values not only highlights the need for standardized protocols to ensure consistent clinical interpretations but also underscores the necessity of maintaining the integrity of patient care across various health systems. Establishing standardized cutoff values could help harmonize treatment protocols and ensure that decisions regarding the initiation of treatment are based on robust and universally accepted criteria, thereby safeguarding patient health while optimizing resource use within healthcare systems. It would also reduce variability in healthcare quality, ensuring that all individuals receive the same standard of care regardless of the institution responsible for their screening and subsequent treatment [23].

Our study also highlights significant issues regarding the specificity of the testing methods currently employed in NBS, particularly MS/MS. This method demonstrated a high rate of false positives for disorders such as 3HMG/3MCC and IVA. The challenges posed by this lack of specificity underscore a perennial issue in medical diagnostics: the delicate balance between sensitivity and specificity. High sensitivity is crucial to ensure that no potential cases are missed, thus reducing the risk of untreated conditions leading to severe outcomes. However, the disadvantage of high sensitivity is often lower specificity, which can lead to a higher rate of false positives. These false positives are not merely statistical errors but can have real-world implications, including unnecessary confirmatory tests, increased healthcare costs, and significant emotional distress for families as they cope with the potential diagnosis of a serious health condition in their newborn. To address these challenges, refinement of testing protocols is necessary. Incorporating second-tier tests could serve as a solution in which initial positive results are followed by more specific tests to confirm the diagnosis, thereby reducing the burden of false positives. Alternatively, enhancing the specificity of the initial screening methodologies may also be beneficial. This could involve adjusting the thresholds for positive results or employing advanced technologies to discriminate between similar metabolites more effectively. The advancement and implementation of UPLC coupled with MS/MS has facilitated the development of novel assays for biomarkers of metabolic diseases, enhancing both the diagnosis and therapeutic monitoring of these disorders [24].

Understanding the distribution patterns is essential to develop public health strategies tailored to the needs of specific populations, thereby enhancing the effectiveness of interventions and optimizing health outcomes. Such insights are particularly valuable for designing preventive measures and educational programs that address the specific needs of populations at higher risk of certain metabolic disorders. For instance, if a metabolic disorder is found to be significantly more prevalent in a particular city, local healthcare providers can be alerted to this trend, ensuring that they are better prepared to promptly diagnose and manage cases. Additionally, public health initiatives could focus on the environmental factors contributing to the higher incidence of the disorder in that area, potentially mitigating the risk factors before they affect newborns.

## 5. Conclusions

In conclusion, the differing prevalence of metabolic disorders across Saudi Arabian cities emphasizes the need for additional genetic studies to clarify the factors influencing newborn metabolic disorders linked to C5 acylcarnitine and related dicarboxylic acylcarnitines. Furthermore, variability in detection cutoffs across healthcare institutions highlights the critical need for standardized screening procedures to ensure accurate and consistent diagnoses, optimizing patient care and resource allocation. Future research should focus on refining diagnostic techniques and developing standardized protocols to ensure equitable and effective care for all infants.

## Figures and Tables

**Table 1:** Positive Predictive Value of Newborn Screening for Metabolic Disorders.

	True Positive (TP)	False Positive (FP)	Positive Predictive Value (PPV) %
3HMG/3MCC	149	99	60.08
BKT	4	3	62.50
GA1	13	4	76.47
IVA	99	83	54.40

**Table 2:** Comparison of cutoff values for primary metabolites.

Analyte	Cutoff	Hospital
C5	>0.7	National Guard Health Affairs, Riyadh
C5DC	>0.38	National Guard Health Affairs, Riyadh
C5OH	>0.5	National Guard Health Affairs, Riyadh
C5	>0.8	Prince Sultan Military Medical City
C5DC	>0.35	Prince Sultan Military Medical City
C5OH	>0.9	Prince Sultan Military Medical City
C5	>0.7	King Faisal Specialist Hospital
C5DC	>0.35	King Faisal Specialist Hospital
C5OH	>0.8	King Faisal Specialist Hospital
C5	>0.7	Public Health Authority
C5DC	>0.6	Public Health Authority
C5OH	>0.8	Public Health Authority

## Data Availability

All the data generated or analyzed in the current study are included in this article.

## Supplementary Materials

The following supporting information can be downloaded at: https://www.mdpi.com/article/10.3390/ijns11020036/s1, Figure S1: Gas chromatography/mass spectrometry (of) with PHL for IVA. The chromatogram represents the metabolites of IVA including 3-hydroxyisovaleric (3HIA) acid retention time (9.80 min) and isovalerylglycine (IVG) with a retention time (17.320 min); Figure S2: Gas chromatography/mass spectrometry (of) with PHL for GA I. The chromatogram represents the metabolites of GA I, including glutaric acid retention time (14.42 min), 3-hydroxyglutaric acid retention time (18.95 min), and glutaconic acid retention time (15.518 min). Figure S3: Gas chromatography/mass spectrometry (GC/MS) in PHL patient for 3HMG. The chromatogram represents the metabolite for 3HMG including 3-hydroxyisovaleric acid (3HIV-A) retention time (9.80 min), 3-methylglutaconic acid (3MGC-A) retention time (15.518 min); Figure S4: Gas chromatography/mass spectrometry (GC/MS) in patients with PHL and 3MCC. The chromatogram represents the metabolites of 3MCC, including 3-methylcrotonyl-glycine (3-MCG) with a retention time (18.448 min),3-hydroxyisovaleric acid (3-HIVA) with a retention time (9.80 min); Figure S5: Gas chromatography/mass spectrometry (GC/MS) for BKT in patients with PHL. The chromatogram represents the metabolites of BKT including as 3 hydroxybutyric acid retention time (8.491 min),2 methyl-3 hydroxybutyric acid retention time (9.582 min), and tiglylglycine (18.2 min) retention times. Table S1: Sociodemographic for disorders, city and gender.
