# Supplementary material for: Characterization of C5 Acylcarnitines and Related Dicarboxylic Acylcarnitines in Saudi Newborns: Screening, Confirmation, and Cutoff Variation
Source: Int J Neonatal Screen. 2025 May 12;11(2):36. doi: 10.3390/ijns11020036 (PMC12101313; doi:10.3390/ijns11020036)
Supplement: Supplementary file 1 [file IJNS-11-00036-s001.zip › Supplementary Data .pdf]

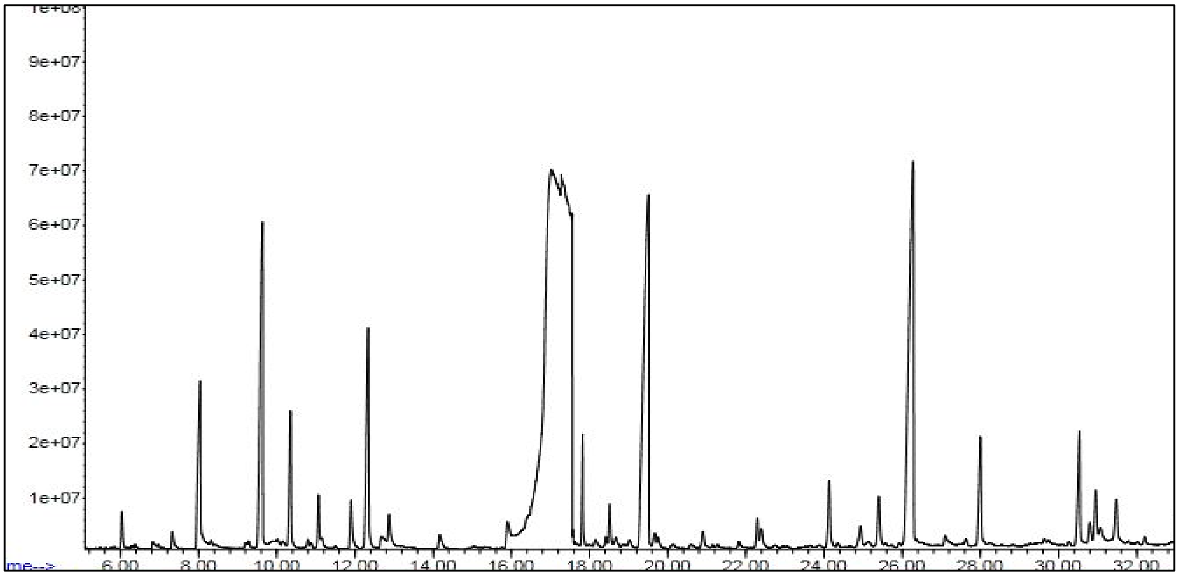

Figure S1. Gas chromatography/mass spectrometry (of) with PHL for IVA. The chromatogram represents the metabolites of IVA including 3-hydroxyisovaleric (3HIA) acid retention time (9.80 min) and and isovalerylglycine (IVG) with a retention time (17.320 min).

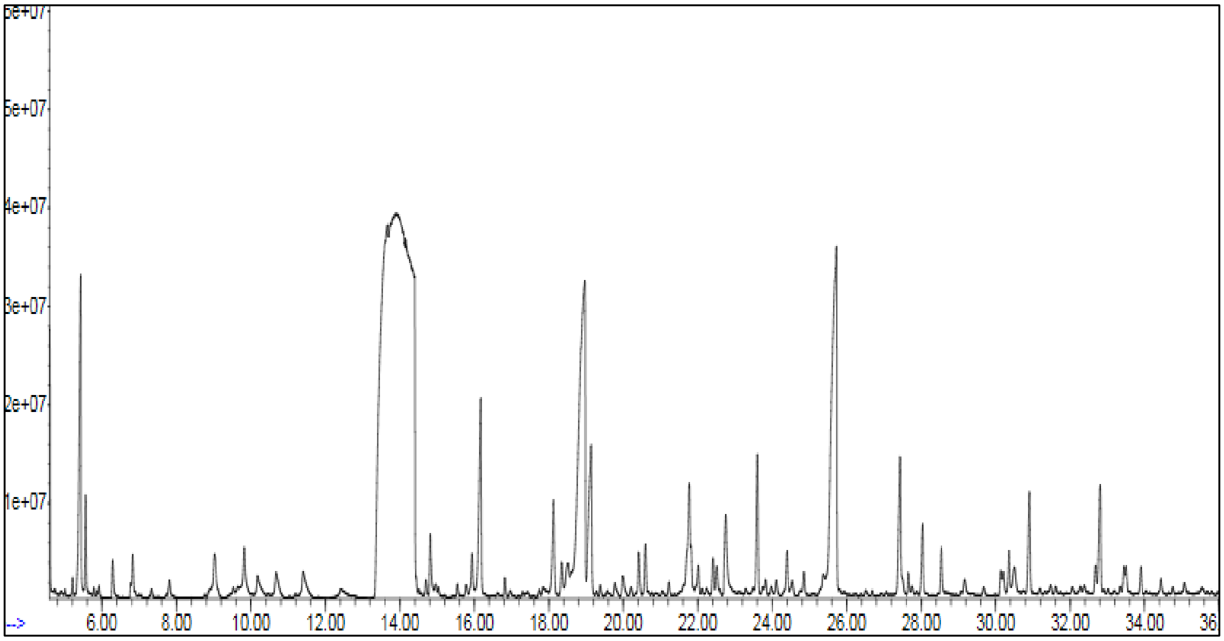

Figure S2. Gas chromatography/mass spectrometry (of) with PHL for GA I. The chromatogram represents the metabolites of GA I, including glutaric acid retention time (14.42 min), 3-hydroxyglutaric acid retention time (18.95 min), and glutaconic acid retention time (15.518 min).

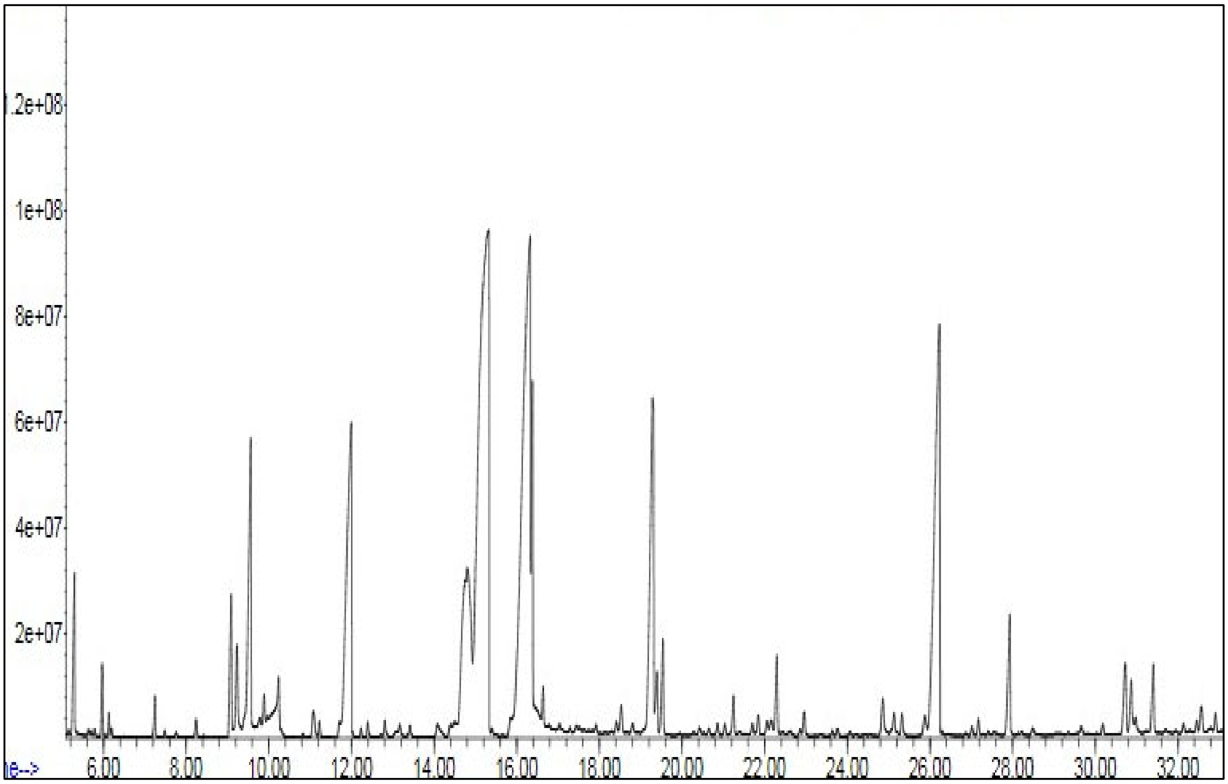

Figure S3. Gas chromatography/mass spectrometry (GC/MS) in PHL patient for 3HMG. The chromatogram represents the metabolite for 3HMG including 3-hydroxyisovaleric acid (3HIV-A) retention time (9.80 min), 3-methylglutaconic acid (3MGC-A) retention time (15.518 min).

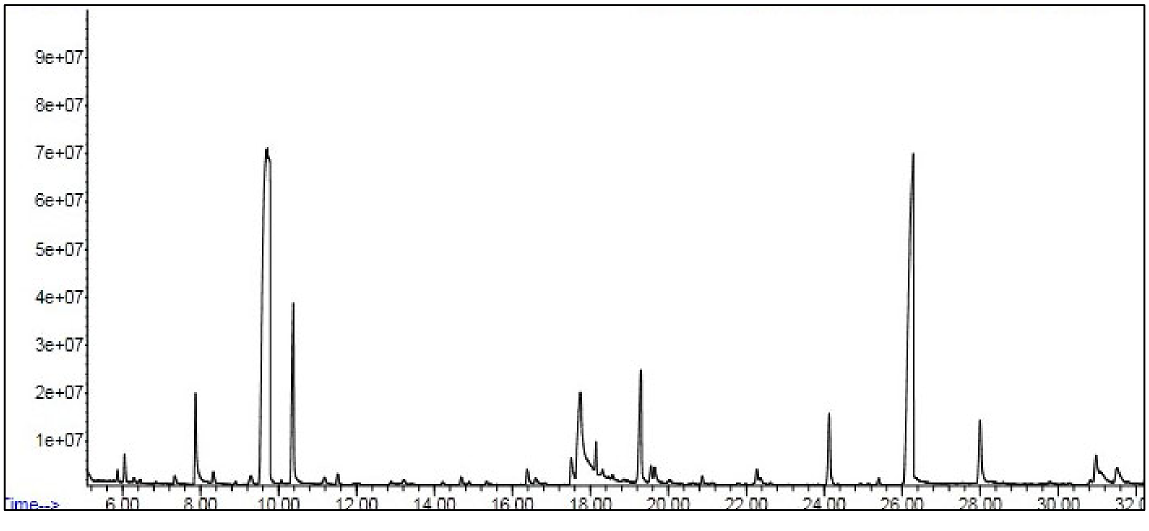

Figure S4. Gas chromatography/mass spectrometry (GC/MS) in patients with PHL and 3MCC. The chromatogram represents the metabolites of 3MCC, including 3-methylcrotonyl-glycine (3-MCG) with a retention time (18.448 min),3-hydroxyisovaleric acid (3-HIVA) with a retention time(9.80 min).

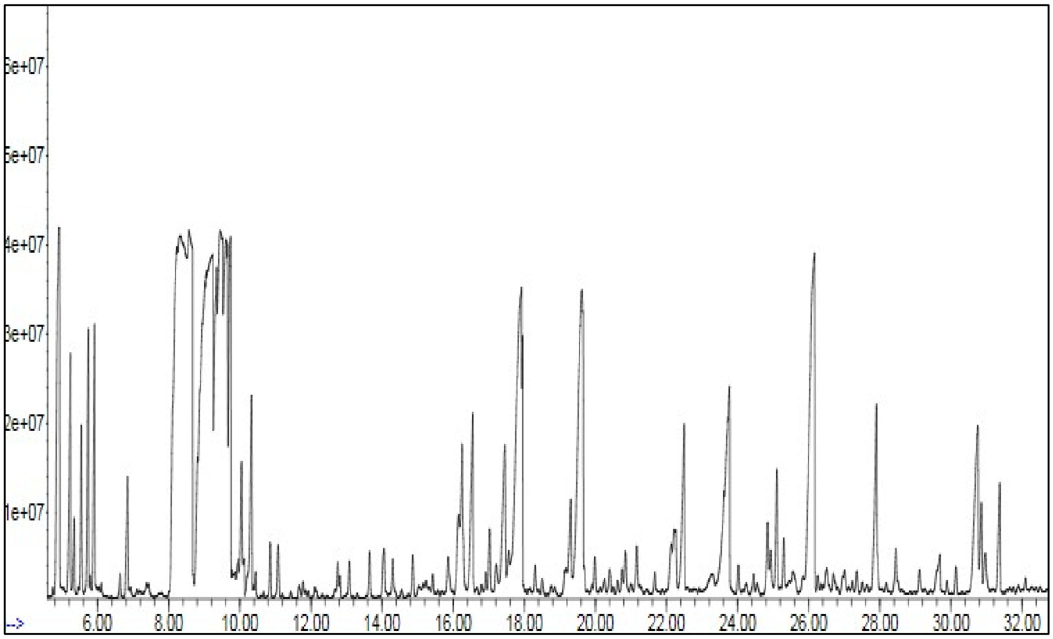

Figure S5. Gas chromatography/mass spectrometry (GC/MS) for BKT in patients with PHL. The chromatogram represents the metabolites of BKT including as 3 hydroxybutyric acid retention time (8.491 min),2 methyl – 3 hydroxybutyric acid retention time (9.582 min), and tiglylglycine (18.2 min) retention times.

Table S1. Sociodemographic for Disorders, city and Gender.

Disorder	City	FEMALE	MALE
3HMG/3MCC	ABHA	1	3
3HMG/3MCC	AL BAHA	5	4
3HMG/3MCC	AL Madinah AL Munawwarah	9	6
3HMG/3MCC	AL TAIF	4	8
3HMG/3MCC	ASEER	5	9
3HMG/3MCC	Al Jouf	3	2
3HMG/3MCC	Al-Ahsa	20	16
3HMG/3MCC	DAMMAM	5	5
3HMG/3MCC	Hafar Al-Batin	0	1
3HMG/3MCC	Hail	0	1
3HMG/3MCC	JAZAN	17	17
3HMG/3MCC	Jeddah	6	10
3HMG/3MCC	MAKKAH	11	13
3HMG/3MCC	Najran	1	0
3HMG/3MCC	Northern border region	0	2
3HMG/3MCC	Qassim	14	13
3HMG/3MCC	Qatif	9	1
3HMG/3MCC	RIYADH	12	13
3HMG/3MCC	Tabuk	0	2
BKT	AL BAHA	1	0
BKT	ASEER	4	0
BKT	JAZAN	0	1
BKT	Jeddah	2	0
GA1	ABHA	1	0
GA1	AL Madinah AL Munawwarah	3	0
GA1	Al-Ahsa	2	0
GA1	DAMMAM	1	0
GA1	JAZAN	1	2
GA1	Jeddah	0	1
GA1	Qassim	1	1

**Disorder**	**City**	**FEMALE**	**MALE**
GA1	RIYADH	2	1
GA1	Tabuk	0	1
IVA	ABHA	0	2
IVA	AL BAHA	3	2
IVA	AL Madinah AL Munawwarah	2	2
IVA	AL TAIF	6	2
IVA	ASEER	3	2
IVA	Al Jouf	0	5
IVA	Al-Ahsa	4	3
IVA	DAMMAM	8	4
IVA	Hafar Al-Batin	5	6
IVA	Hail	6	0
IVA	JAZAN	0	2
IVA	Jeddah	3	0
IVA	MAKKAH	24	24
IVA	Najran	4	2
IVA	Northern border region	5	1
IVA	Qassim	4	7
IVA	Qatif	9	1
IVA	RIYADH	16	15
